# Circulating sCD14 Is Associated with Virological Response to Pegylated-Interferon-Alpha/Ribavirin Treatment in HIV/HCV Co-Infected Patients

**DOI:** 10.1371/journal.pone.0032028

**Published:** 2012-02-21

**Authors:** Giulia Marchetti, Paola Nasta, Francesca Bai, Francesca Gatti, Giusi Maria Bellistrì, Camilla Tincati, Federica Borghi, Giampiero Carosi, Massimo Puoti, Antonella d'Arminio Monforte

**Affiliations:** 1 Department of Medicine, Surgery and Dentistry, Clinic of Infectious Diseases and Tropical Medicine, University of Milan, San Paolo Hospital, Milan, Italy; 2 Institute of Infectious and Tropical Diseases, University of Brescia, Brescia, Italy; 3 Department of Infectious Diseases, A.O. Ospedale Niguarda Cà Granda, Milan, Italy; University of Pittsburgh Center for Vaccine Research, United States of America

## Abstract

**Objectives:**

Microbial translocation (MT) through the gut accounts for immune activation and CD4+ loss in HIV and may influence HCV disease progression in HIV/HCV co-infection. We asked whether increased MT and immune activation may hamper anti-HCV response in HIV/HCV patients.

**Methods:**

98 HIV/HCV patients who received pegylated-alpha-interferon (peg-INF-alpha)/ribavirin were retrospectively analyzed. Baseline MT (lipopolysaccharide, LPS), host response to MT (sCD14), CD38+HLA-DR+CD4+/CD8+, HCV genotype, severity of liver disease were assessed according to Early Virological Response (EVR: HCV-RNA <50 IU/mL at week 12 of therapy or ≥2 log_10_ reduction from baseline after 12 weeks of therapy) and Sustained Virological Response (SVR: HCV-RNA <50 IU/mL 24 weeks after end of therapy). Mann-Whitney/Chi-square test and Pearson's correlation were used. Multivariable regression was performed to determine factors associated with EVR/SVR.

**Results:**

71 patients displayed EVR; 41 SVR. Patients with HCV genotypes 1–4 and cirrhosis presented a trend to higher sCD14, compared to patients with genotypes 2–3 (p = 0.053) and no cirrhosis (p = 0.052). EVR and SVR patients showed lower levels of circulating sCD14 (p = 0.0001, p = 0.026, respectively), but similar T-cell activation compared to Non-EVR (Null Responders, NR) and Non-SVR (N-SVR) subjects. sCD14 resulted the main predictive factor of EVR (0.145 for each sCD14 unit more, 95%CI 0.031–0.688, p = 0.015). SVR was associated only with HCV genotypes 2–3 (AOR 0.022 for genotypes 1–4 vs 2–3, 95%CI 0.001–0.469, p = 0.014).

**Conclusions:**

In HIV/HCV patients sCD14 correlates with the severity of liver disease and predicts early response to peg-INF-alpha/ribavirin, suggesting MT-driven immune activation as pathway of HIV/HCV co-infection and response to therapy.

## Introduction

Up to 25–50% of HIV-positive patients are co-infected with HCV [1], [2]. In the setting of HIV disease, clinical manifestations of HCV infection are severe, leading to rapid liver damage and cirrhosis [3]–[8]. Indeed, liver-related mortality is a leading cause of death in HIV-infected population [9]–[11].

Following current standard treatment of HCV infection with pegylated-interferon-alpha (peg-INF-alpha) plus ribavirin, HCV mono-infected patients reach a sustained virologic response (SVR) with eradication of the virus in 50–80% of cases; conversely, the rate of SVR is substantially lower in HIV/HCV co-infected subjects [12], [13]. Despite several factors have been associated with response to anti-HCV therapy, the determinants of successful outcome of full course peg-INF-alpha/ribavirin therapy are still scantily defined [14]–[18].

No HCV-RNA decrease ≥2 log_10_ after 3 months of treatment, that is called “null response” (NR), is associated with a >95% probability of no sustained response and has recently been associated with a very low response to retreatment with triple therapies including peg-INF-alpha/ribavirin/anti-HCV protease inhibitors [18]–[22]. Conversely, HCV-RNA decrease ≥2 log_10_ after 3 months of treatment, i.e. Early Virologic Response (EVR), has a positive predictive value on SVR of 72–84% [19], [20].

During HIV infection, a dramatic depletion of CD4+ T cells in the gut occurs [23], leading to the speculation that injury to the immune component of the gastrointestinal mucosal surface may induce increased translocation of microbial products in the systemic circulation, particularly LPS, despite a direct cause-effect nexus between gut mucosa disruption and microbial translocation has not been established yet [24]. Increased levels of circulating LPS correlate with immune activation, in turn playing a key role in the pathogenesis of disease progression and CD4+ lymphopenia [24]–[26].

Recently, the role of LPS has also been analyzed as a possible underlying cause of liver disease. Subjects with HCV mono-infection display a high degree of microbial translocation (MT) and elevated LPS levels are strongly associated with the severity of liver disease [27], [28]; likewise, augmented plasma LPS also features non-alcoholic fatty liver disease [29]–[31]. Furthermore, in animal models LPS appears to accelerate liver fibrosis, both directly by increasing hepatic fibrosis and stimulating liver Kupffer cells, and indirectly by enhancing immune activation [32], [33].

Given the importance of early predictive parameters of therapeutic efficacy, and above all, of EVR, in order to limit the frequent side effects of drug exposure in patients unlikely to benefit from treatment, we sought to investigate whether measures of MT before the initiation of anti-HCV treatment correlates with EVR and/or SVR to HCV therapy in HIV/HCV co-infected patients.

## Materials and Methods

### Study design and population

We retrospectively analyzed chronic HIV/HCV co-infected patients attending the Institute of Infectious and Tropical Diseases, University of Brescia at “Spedali Civili”, Brescia and the Clinic of Infectious Diseases, University of Milan at “San Paolo” Hospital in Milan, Italy between January 2005 and December 2009. This study was approved by the Ethic Committees of the University of Brescia at “Spedali Civili”, Brescia and of the University of Milan at “San Paolo” Hospital. Each patient gave written informed consent to blood collection for research purpose, upon approval of the form by Local Ethic Committee.

Inclusion criteria were to have initiated at least one dose of a treatment including peg-INF-alpha 2a and 2b subcutaneously once weekly (180 mcg or 1.5 mcg/Kg) plus daily weight-dosed ribavirin (1000 mg/day for a pretreatment weight <75 Kg or 1200 mg/day for weight ≥75 Kg).

According to the virological response at week 12 of treatment, patients were considered: (i) Early Virological Responders (EVR), undetectable serum HCV-RNA (<50 IU/mL) or ≥2 log_10_ reduction from baseline after 12 weeks of therapy; (ii) Null Responders (NR), serum HCV-RNA ≥50 IU/mL and <2 log_10_ reduction from baseline.

SVR was defined as undetectable serum HCV-RNA (<50 IU/mL) 24 weeks after the end of a full course of 48 or 72 weeks of anti-HCV treatment, according to genotype. Baseline was defined as time of anti-HCV treatment introduction (within one month before the beginning of therapy). Subjects who were lost at follow-up before week 12 or before the time point established for SVR assessment were considered NR patients and Sustained Virological Non Responders (N-SVR patients), respectively.

### Staging of liver disease

The stage of liver disease was determined in a subgroup of patients who underwent percutaneous liver biopsies, or liver stiffness by transient elastography (FibroScan®, Echo Sense Paris, France). Only the examinations with an Interquartile Range below 30% of the median value and a success rate of acquisition above 60% were considered [34]. The Ishak-modified Knodell score system (2000) was used to assess necro-inflammatory activity and fibrosis. Advanced fibrosis was defined as Knodell score F3–4 on liver biopsy or liver stiffness greater than 12.5 kPa on Fibroscan. Hepatic cirrhosis was defined as the presence of a histopathological diagnosis, a liver surface nodularity assessed by ultrasound or a liver stiffness greater than 17 kPa.

### MT and immune activation analysis

LPS and sCD14 plasma levels were quantified on fasting samples with commercially available kits (Limulus Amebocyte Assay, Cambrex, Italy; ELISA assay, R&D, Milan, Italy) as previously described [25].

Activated HLA-DR+CD38+CD4+/CD8+ were evaluated on thawed PBMCs: HLA-DR fluorescein isothiocyanate (FITC), CD38 phycoerytrin (PE), CD4 PerCPcyanin 5.5 (PerCPCy 5.5), CD8 PerCPcyanin 5.5 (PerCPCy 5.5) (Becton Dickinson, San Josè, CA, USA). The following combinations were used: CD4/HLA-DR/CD38, CD8/HLA-DR/CD38. The proportion of HLA-DR+CD38+ was calculated on gated CD4+ and CD8+ T-cells.

### Statistical analysis

Baseline differences between patients' groups were assessed using Mann-Whitney non-parametric U test and Chi-squared test for continuous and categorical variables, respectively. Comparison of MT and immune activation between patients with HCV genotypes 1–4 *versus* 2–3, absence-moderate *versus* advanced fibrosis, cirrhosis *versus* not cirrhosis, EVR *versus* NR and SVR *versus* N-SVR patients were evaluated by Mann-Whitney U test. Multivariable logistic regression model was used to identify baseline parameters that were independently associated with EVR or SVR. Multivariate analysis was performed in 65/98 patients for whom all the clinical, epidemiological and biological parameters under study were available. Factors considered in the logistic regression model were age, sex, baseline CD4+ T cell count, HCV genotypes, HCV-RNA, fibrosis, cirrhosis and MT. Analyses were performed using SPSS software (version 18).

## Results

### Study population

Ninety-eight HIV/HCV co-infected patients undergoing anti-HCV treatment were enrolled. Table 1 shows the clinical, epidemiological, and laboratory data of the patients under study. 71 patients (72.5%) achieved EVR; 27 subjects (27.5%) displayed NR. Eight out of 98 patients stopped HCV therapy before 12 weeks and were lost at follow up (Table 1).

**Table 1:** Baseline demographic and immuno-virological characteristics of study population.

Characteristics	Patients (98)	EVR (71)	NR (27)	p (EVR vs NR)
Age, years *	44 (41–46)	44 (41–46)	43 (40–46)	0.461
Gender, male °	81 (82.7)	57 (80.3)	24 (88.9)	0.385
Time since 1^st^ diagnosis of HIV, (years) *	18 (13–20)	18 (13–21)	19 (12–20)	0.466
Duration of cART, (years) *	10 (8–13)	10 (8–14)	10 (5–12)	0.592
cART °				0.845
Naive	2 (2)	1 (1.5)	1 (3.7)	
NNRTI+NRTI	14 (14.3)	11 (15.5)	3 (11.1)	
NRTI+PI	74 (75.5)	53 (74.6)	21 (77.8)	
Other	8 (8.2)	6 (8.4)	2 (7.4)	
Exposure cathegories °				0.778
MSM	3 (3)	2 (2.8)	1 (3.7)	
WSM	8 (8.2)	5 (7)	3 (11.1)	
IDUs	87 (88.8)	64 (90.2)	23 (85.2)	
Previous AIDS diagnosis °	24/93 (25.8)	14/67 (20.9)	10/26 (38.5)	0.082
CD4+ T cells/µL nadir *	152 (67.5–251.5)	173 (106.5–258.5)	100 (36–198)	0.045
Baseline CD4+ T cells/µL *	430 (321.5–567)	433 (321–555)	428 (300–687)	0.659
CD4+ T cells % *	26.9 (21.9–33)	27.6 (22.1–33.7)	25 (19.9–29.1)	0.274
CD8+ T cells/µL *	601.5 (467.5–939)	626 (429–900)	589 (492–1132)	0.556
CD8+ T cells % *	40.9 (33.9–47)	40.3 (33.6–47)	41.2 (34.1–48.5)	0.578
Baseline HIV-RNAlog_10_ cp/mL *	1.7	1.7	1.7	0.893
Time to HIV-RNA<40 cp/mL (months)*	41 (20–69)	41 (18–72.2)	43 (20–58.7)	0.569
HCV genotypes °				0.0001
1–4	45 (45.9)	23 (32.4)	22 (81.5)	
2–3	53 (54.1)	48 (67.6)	5 (18.5)	
Baseline HCV-RNAlog_10_ IU/mL *	5.5 (4.9–6.03)	5.6 (4.8–5.8)	5.8 (5.2–6.5)	0.015
Cirrhosis °	30/94 (31.2)	18/69 (26.1)	12/25 (48)	0.044
Advanced fibrosis °	43/89 (48.3)	29/66 (43.9)	14/23 (60.9)	0.162
HBV infection (HBsAg+) °	7 (7.1)	4 (5.6)	3 (11.1)	0.390
Serum AST (UI/L) *	109.8 (42.2–94)	67 (42–94)	71 (42–103)	0.972
Serum ALT (UI/L) *	89 (55–137.2)	97 (51–145)	85 (58–110)	0.548
BMI *	22.9 (21.2–25.1)	22.9 (21.1–25.4)	22.8 (21.6–25.6)	0.320
Glycemia (mg/dL) *	89 (83–97.5)	90 (83.7–101.7)	88 (79.2–96.5)	0.492
Insulinemia (UI/L) *	12.9 (9.4–20.1)	13 (9.4–20.6)	11 (8.1–18.2)	0.526
Alcol (gr/die) *	0 (0–11)	0 (0–10)	0 (0–5)	0.755
HOMA index *	2.6 (1.7–4.1)	2.5 (1.7–4)	2.9 (1.6–4.3)	0.972

**LEGEND.** Data are presented as *median, (IQR) and °absolute number, (%). Differences between groups were compared by *Mann Whitney U test and °χ2 test. EVR, Early Virological Response: undetectable serum HCV-RNA (<50 IU/mL) or ≥2 log_10_ reduction from baseline after 12 weeks of therapy; NR, Null Responders: serum HCV-RNA ≥50 IU/mL and <2 log_10_ reduction from baseline. cART, Combination Antiretroviral therapy; NRTI, Nucleoside Reverse Transcriptase Inhibitors; NNRTI, Non Nucleoside Reverse Transcriptase Inhibitors; PI, Protease Inhibitors. MSM, men who have sex with men; WSM, women who have sex with men; IDUs, injection drug users. HCV, hepatitis C virus; HBV, hepatitis B virus; HBsAg, hepatitis B surface antigen. AST, aspartate aminotransferase; ALT, alanine aminotransferase. BMI, Body Mass Index. HOMA index, Homeostatic Model Assessment index.

Patients' groups were comparable for demographic, HIV related variables, and metabolic parameters (Table 1).

### MT and HCV related parameters

Plasma LPS and sCD14 levels were assessed in a subgroup of unselected patients for whom plasma samples were available (65 and 91 samples, respectively). Baseline characteristics of the 65 and 91 patients for whom LPS and sCD14 measurements were performed were comparable to the main population under study.

**Figure 1:**
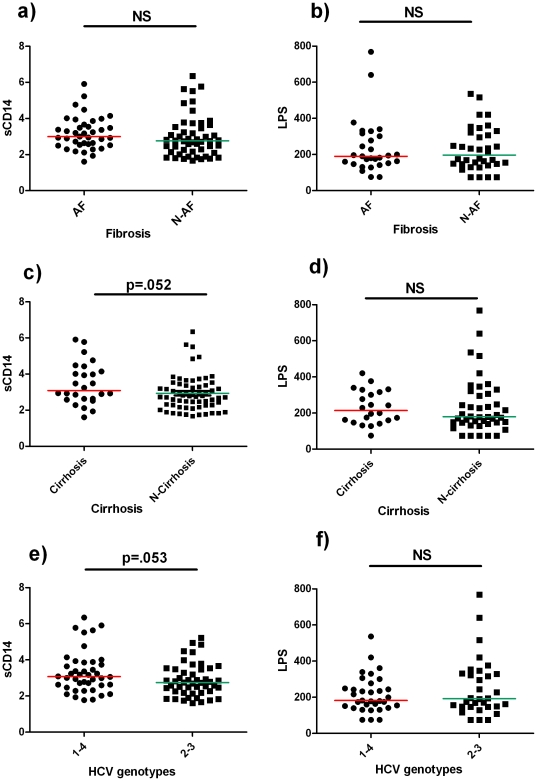
Higher microbial translocation is associated with HCV genotypes 1–4 and cirrhosis. **a**)**-b**) sCD14 and LPS were compared between patients with advanced fibrosis (AF) and non advanced fibrosis (N-AF). **c**)**-d**) sCD14 and LPS were compared between patients with cirrhosis and absence of cirrhosis (N-Cirrhosis). **e**)**-f**) sCD14 and LPS were compared between patients with HCV genotypes 1–4 and genotypes 2–3. Each point represents the value from one subject's plasma. sCD14 and LPS were measured in plasma samples; sCD14 µg/mL, LPS pg/mL. AF = advanced fibrosis – N-AF = non advanced fibrosis. p-values were assessed by Mann Whitney U test. p>0.05 was considered non significant (NS).

Compared to initial-moderate hepatic disease, the presence of advanced hepatic fibrosis was not associated with higher sCD14 levels [median sCD14, patients with advanced hepatic fibrosis: 3, (IQR 2.53–3.66) - patients with initial-moderate hepatic disease: 2.77, (IQR 2.25–3.48), p = 0.128], (Figure 1a).

Patients with cirrhosis were characterized by a trend to higher sCD14 plasma levels, albeit not reaching statistical significance [median sCD14, patients with cirrhosis: 3.09, (IQR 2.62–4.22) - patients without cirrhosis: 2.79, (IQR 2.29–3.44), p = 0.052], (Figure 1c).

Similarly, patients with HCV genotypes 1–4 tended to display higher sCD14 [median sCD14, HCV genotype 1–4: 3.07, (IQR 2.56–3.86) - HCV genotype 2–3: 2.75, (IQR 2.2–3.48), p = 0.053; (Figure 1e). Aiming to further investigate the association between circulating sCD14 and HCV genotype, we performed 2 sensitivity analyses: i) excluding patients with advanced fibrosis; ii) excluding patients with cirrhosis. Most interestingly, patients with genotypes 1–4 still displayed significantly higher circulating sCD14 according to both i) and ii) analyses [i): median sCD14, HCV genotype 1–4: 3.1, (IQR 2.6–3.7) - HCV genotype 2–3: 2.6, (IQR 1.9–2.8), p = 0.022; ii): median sCD14, HCV genotype 1–4: 3.07, (IQR 2.6–3.7) - HCV genotype 2–3: 2.63, (IQR 2.1–3.2), p = 0.028].

As shown in Figure 1b, d and f, no significant association was shown between LPS levels and advanced fibrosis, cirrhosis and HCV genotypes.

### T cell activation and HCV related parameters

We studied HLA-DR and CD38 expression on CD4+ and CD8+ T cells in an unselected subgroup of 53 patients for whom PBMC samples were available. Baseline characteristics of this subgroup of patients were comparable to the main population under study.

**Figure 2:**
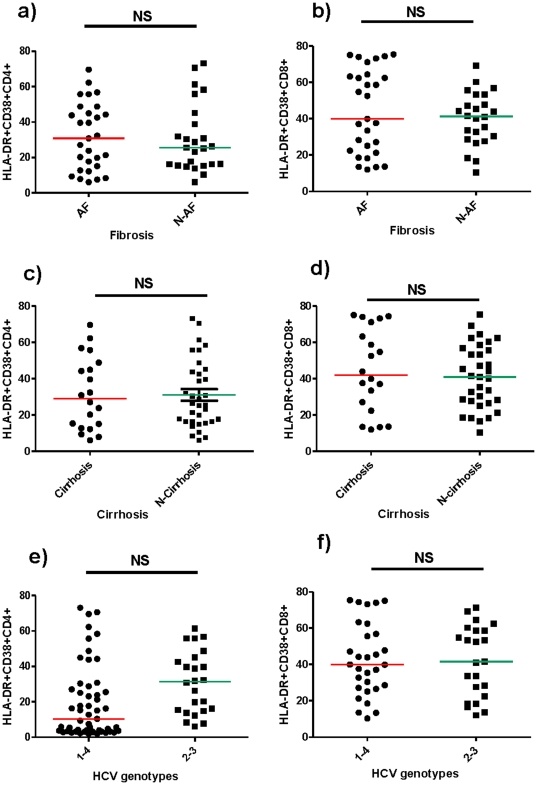
Activated HLA-DR+CD4+ and CD8+ T-cells according to liver fibrosis, cirrhosis and HCV genotypes. **a**)**-b**) Activated HLA-DR+CD4+ and CD8+ T-cells were compared between patients with advanced fibrosis (AF) and non advanced fibrosis (N-AF). **c**)**-d**) Activated HLA-DR+CD4+ and CD8+ T-cells were compared between patients with cirrhosis and absence of cirrhosis (N-Cirrhosis). **e**)**-f**) Activated HLA-DR+CD4+ and CD8+ T-cells were compared between patients with HCV genotypes 1–4 and genotypes 2–3. Each point represents the value from one subject's plasma. Activated HLA-DR+CD4+ and CD8+ T-cells % values are presented. AF = advanced fibrosis – N-AF = non advanced fibrosis. p-values were assessed by Mann Whitney U test. p>0.05 was considered non significant (NS).

Comparable activated T cell numbers were shown in patients with advance fibrosis compared to those with absent or initial-moderate fibrosis [HLA-DR+/CD38+/CD4+, advanced fibrosis: 30.8, (IQR 13.9–46.8) - absence or initial-moderate fibrosis: 25.8, (IQR 16.2–43.5), p = 0.957; HLA-DR+/CD38+/CD8+, advanced fibrosis: 39.9, (IQR 21.8–63.8) - absence or initial-moderate fibrosis: 41.01, (IQR 28.6–53.2), p = 0.513] (Figure 2a, b) and in patients with cirrhosis compared to subjects not displaying cirrhosis [HLA-DR+/CD38+/CD4+, cirrhosis: 28.9, (IQR 13.3–47.8) – absence of cirrhosis: 25.8, (IQR 16.2–44.1), p = 0.971; HLA-DR+/CD38+/CD8+, cirrhosis: 41.9, (IQR 23.6–69.2) – absence of cirrhosis: 41.1, (IQR 27.1–56.2), p = 0.62] (Figure 2c, d).

Similar activated CD4+ and CD8+ T cell proportions were shown by patients with genotypes 1–4 and patients with genotypes 2–3 [HLA-DR+/CD38+/CD4+, genotypes 1–4: 25.26, (IQR 15.94–45.87) - genotypes 2–3: 31.34, (IQR 14.94–44.47), p = 0.986; HLA-DR+/CD38+/CD8+, genotypes 1–4: 39.95, (IQR 26.94–58.21) – genotypes 2–3: 41.53, (IQR 22.39–58.7), p = 0.914] (Figure 2e, f).

### Immune activation and MT according to EVR to anti-HCV treatment

To address the association of peripheral T cell activation with the outcome of peg-INF-alpha/ribavirin anti-HCV treatment, we also analyzed HLA-DR+CD38+CD4+ and CD8+ T cells in EVR and NR patients.

**Figure 3:**
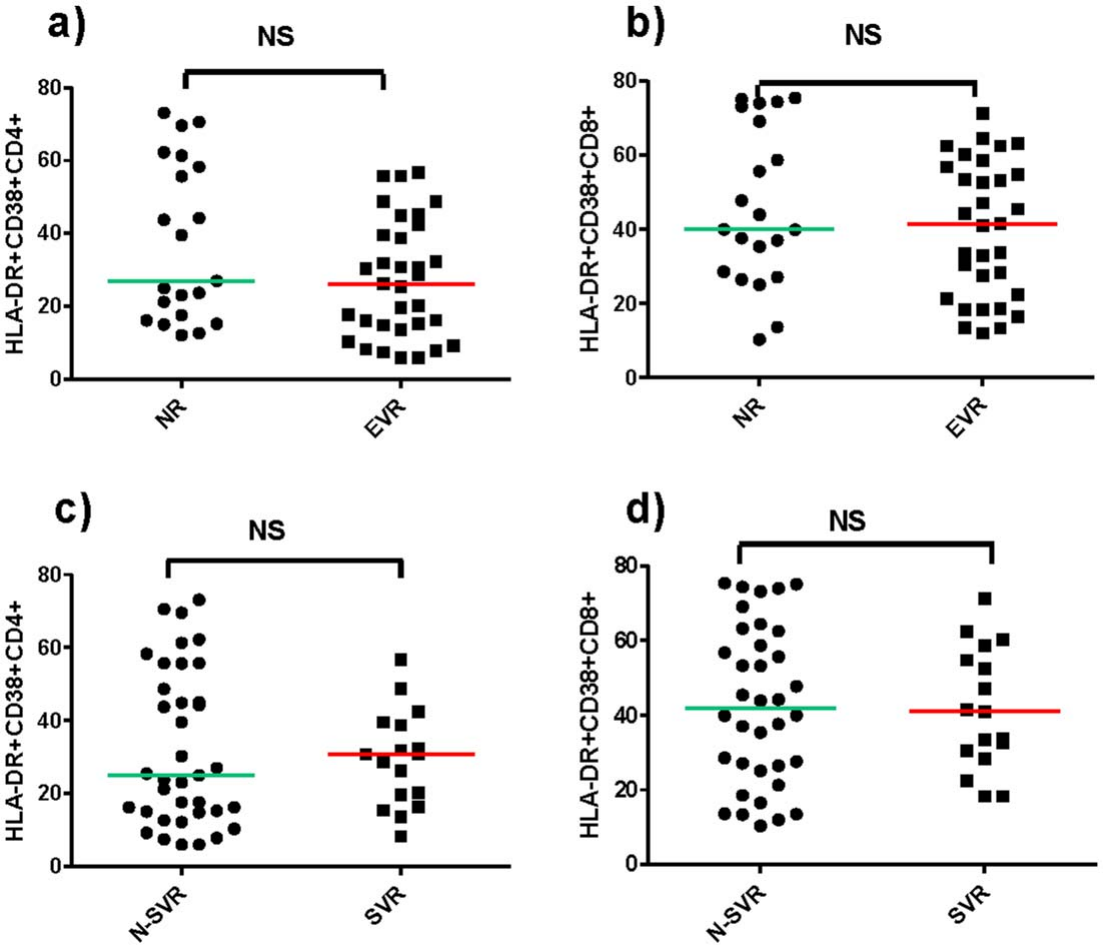
Activated HLA-DR+CD4+ and CD8+ T-cells according to EVR and SVR. **a**)**-b**) Activated HLA-DR+CD4+ and CD8+ T-cells were compared between patients with early virological response [EVR, i.e. undetectable serum HCV-RNA (<50 IU/mL) or ≥2 log_10_ reduction from baseline after 12 weeks of therapy], and Null Responders (NR) (i.e. serum HCV-RNA ≥50 IU/mL and <2 log_10_ reduction from baseline). **c**)**-d**) Activated HLA-DR+CD4+ and CD8+ T-cells were compared between patients with sustained virological response [SVR, i.e. undetectable serum HCV-RNA (<50 IU/mL) 24 weeks after the end of a full course of 48 or 72 weeks of anti-HCV treatment, according to genotype], and N-SVR subjects. Each point represents the value from one subject's plasma. Activated HLA-DR+CD4+ and CD8+ T-cells % values are presented. p-values were assessed by Mann Whitney U test. p>0.05 was considered non significant (NS).

Median HLA-DR+CD38+CD4+ and CD8+ were similar in both groups, (HLA-DR/CD38+/CD4+, EVR 34.1 (IQR 19.3–48.3) – NR 35.4 (IQR 26–66.7), p = 0.097; HLA-DR/CD38+/CD8+, EVR 51.2 (IQR 27.2–65.9) – NR 50.5 (IQR 35.2–76.1), p = 0.309) (Figure 3a, b).

**Figure 4:**
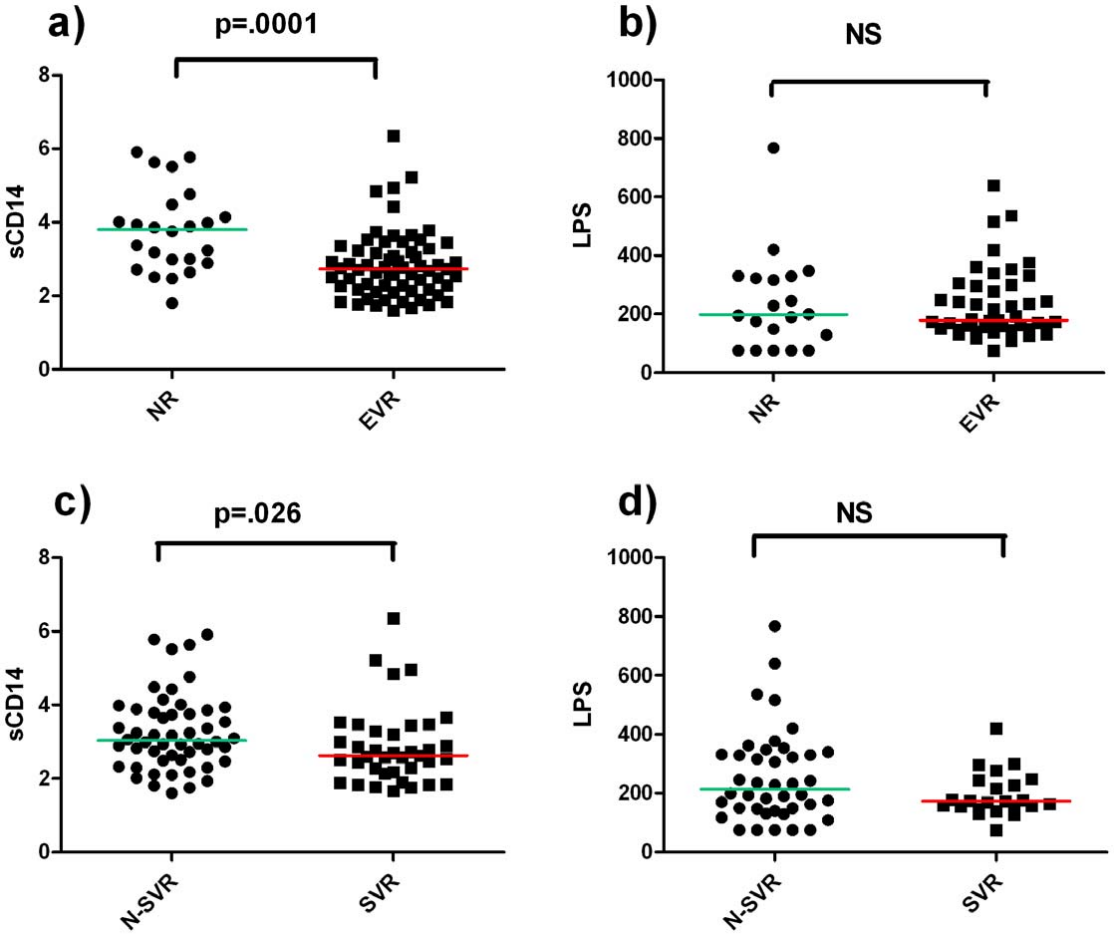
Circulating sCD14 and LPS levels are higher in NR and in N-SVR patients. **a**)**-b**) sCD14 and LPS were compared between patients with early virological response [EVR, i.e. undetectable serum HCV-RNA (<50 IU/mL) or ≥2 log_10_ reduction from baseline after 12 weeks of therapy], and Null Responders (NR) (i.e. serum HCV-RNA ≥50 IU/mL and <2 log_10_ reduction from baseline). **c**)**-d**) sCD14 and LPS were compared between patients with sustained virological response [SVR, i.e. undetectable serum HCV-RNA (<50 IU/mL) 24 weeks after the end of a full course of 48 or 72 weeks of anti-HCV treatment, according to genotype], and N-SVR subjects. Each point represents the value from one subject's plasma. sCD14 and LPS were measured in plasma samples; sCD14 µg/mL, LPS pg/mL. p-values were assessed by Mann Whitney U test. p>0.05 was considered non significant (NS).

In addition, to assess potential interactions between MT and virological response to anti-HCV treatment, baseline LPS and sCD14 levels were compared between EVR subjects and NR (Figure 4a, b). Interestingly, EVR patients presented significantly lower median sCD14 (EVR 2.73, IQR: 2.18–3.25 – NR 3.8, IQR: 2.9–4.4, p = 0.0001) (Figure 4a), despite no differences in median LPS levels between patients (EVR 178.7, IQR: 150.7–298.3 – NR 197.7, IQR: 88.4–327.4, p = 0.865), (Figure 4b).

### Identification of factors associated to EVR to anti-HCV treatment

Given that the host response to MT expressed by sCD14 was lower in EVR patients at baseline, we investigated its association with EVR after adjustment for demographic and HCV-related variables. Table 2 shows results from univariate and multivariate analyses.

**Table 2:** Association between markers of microbial translocation and Early Virological Response to anti-HCV treatment.

	Univariate			Multivariate		
	OR	95%CI	P	AOR	95%CI	p
LPS (pg/mL)	1.000	0.996–1.004	0.934	0.997	0.99–1.004	0.345
sCD14 (µg/mL)	0.419	0.252–0.695	**0.001**	0.145	0.031–0.688	**0.015**
HCV genotypes(1–4 vs 2–3)	0.109	0.037–0.324	**0.0001**	0.233	0.021–2.618	0.238
HCV-RNA(log_10_ IU/mL)	0.409	0.207–0.809	**0.01**	0.789	0.134–4.628	0.793
Fibrosis(advanced vs non advanced)	0.504	0.191–1.327	0.165	0.134	0.005–3.879	0.616
Cirrhosis (yes vs no)	0.382	0.148–0.99	**0.048**	0.185	0.007–4.623	0.304
Nadir CD4+ T cells/µL	1.003	0.999–1.006	0.155	1.007	0.998–1.016	0.134
CD4+ T cells/µL	0.999	0.997–1.002	0.518	0.996	0.990–1.001	0.112
Age, years	1.043	0.948–1.149	0.386	1.134	0.879–1.463	0.333
Sex, male vs female	0.509	0.134–1.934	0.321	0.215	0.007–6.926	0.385

**LEGEND.** LPS, soluble CD14, CD4+ T cells/µL, age, HCV-RNA log_10_ cp/mL for each unit more. sCD14 and LPS were measured in plasma samples; sCD14 µg/mL, LPS pg/mL. Multivariate analysis was performed in 65/98 patients for whom all the clinical, epidemiological and biological parameters under study were available.

OR, odds ratio; AOR, adjusted odds ratio; CI, confidence interval. p>0.05 was considered non significant.

In the univariate analysis, higher sCD14 was associated with a reduced probability of reaching EVR (OR 0.419 for each sCD14 unit more, 95%CI 0.252–0.695, p = 0.001), whereas no association was shown between LPS and EVR (OR 1.000 for each LPS unit more, 95%CI 0.996–1.004, p = 0.934). As expected, HCV genotypes 1–4 (OR 0.109 *versus* HCV genotypes 2–3, 95%CI 0.037–0.324, p = 0.0001), higher HCV viral load (OR 0.409 for each log_10_ IU/mL more, 95%CI 0.207–0.809, p = 0.01), and cirrhosis (OR 0.382 *versus* absence of cirrhosis, 95%CI 0.148–0.99, p = 0.048) were all associated to a decreased chance of EVR (Table 2).

In line with univariate data, the proportion of patients with cirrhosis were more likely to fail EVR (cirrhosis/NR = 48%, cirrhosis/EVR = 26%, p = 0.044); similarly a higher proportion of genotype 1–4 patients did not reach EVR (genotype 1–4/NR = 81%, genotype 1–4/EVR = 32%, p = 0.0001). No differences were shown in EVR/NR according to the presence fibrosis (p = 0.162).

Significantly predictive parameters in the univariate analysis entered the logistic regression model adjusted for age, sex and baseline CD4+ T cell count (Table 2).

Overall, multivariate analysis shown in Table 2 was performed in 65/98 patients for whom both LPS and sCD14 data were available. Baseline characteristics of these 65 patients were comparable to the entire cohort.

In the adjusted model, higher sCD14 levels (AOR 0.145 for each unit more, 95%CI 0.031–0.688, p = 0.015) was confirmed as predictive independent marker of decreased chances of EVR (Table 2). No other demographic or HCV related variable that we studied resulted independently associated with EVR (Table 2).

Similar results were obtained also excluding from the analyses the 8/98 patients who did not reach 12 weeks follow up (data not shown).

### Baseline characteristics of the study population according to SVR

SVR data were available for 88 subjects. Ten patients have not reached the end of the study at the time of analysis.

Overall, 41 patients (46.6%) exhibited SVR. Table 3 shows the clinical, epidemiological, and laboratory data of the patients under study according to SVR. Patients achieving and not achieving SVR were comparable for baseline demographic parameters (Table 3). Indeed, even if baseline CD4+ T cells were similar, SVR subjects presented higher CD4+ nadir and higher current CD8+, compared to N-SVR (median, IQR: nadir CD4+ T cells SVR 212/µL, 120–268 – N-SVR 131/µL, 45–198, p = 0.025; CD8+ T cells SVR 693/µL, 558–1048.5 – N-SVR 540/µL, 383–952, p = 0.030) (Table 3).

**Table 3:** Baseline demographic and immuno-virological characteristics of patients according SVR.

Characteristics	SVR (41)	N-SVR (47)	p
Age, years *	44 (40–47.5)	43 (42–46)	0.997
Gender, male °	33 (80)	40 (85)	0.565
Time since 1^st^ diagnosis of HIV, (years)*	18 (13–22)	18 (15–20)	0.700
Duration of HAART, (years) *	10 (5–14)	11 (9–13)	0.192
HAART °			0.849
naive	1 (2)	1 (2)	
NNRTI+NRTI	7 (17)	5 (11)	
NRTI+PI	30 (73)	37 (79)	
other	3 (7)	4 (8)	
Exposure cathegories°			0.300
MSM	2 (5)	0	
WSM	2 (5)	3 (6)	
IDUs	37 (90)	44 (94)	
Previous AIDS diagnosis °	8/39 (21)	13/44 (30)	0.345
CD4+ T cells/µL nadir *		131 (45.2–198)	0.025
Baseline CD4+ T cells/µL *	467 (326.5–574)	398 (300–582)	0.281
CD4 T cells % *	26.3 (21.5–32.1)	27.5 (20–34)	0.977
CD8 T cells/µL *	693 (558–1048.5)	540 (383–952)	0.030
CD8 T cells % *	41.1 (35.8–49.5)	40.4 (33.6–47)	0.315
Baseline HIV-RNA log_10_ cp/mL *	1.7	1.7	0.934
Time to HIV-RNA <40 cp/mL (mts)*	43 (15–72)	42 (21–69)	0.986
HCV genotypes °			0.0001
1–4	7 (17)	33 (70)	
2–3	36 (88)	14 (30)	
Baseline HCV-RNA log_10_ IU/mL *	5.3 (4.7–5.9)	5.68 (5.3–6.2)	0.014
Cirrhosis °	9/41 (22)	19/44 (43)	0.037
Advanced fibrosis °	15/39 (38)	23/41 (56)	0.114
HBV infection (HBsAg+) °	2 (5)	4 (9)	0.681
Serum AST (UI/L) *	69 (44–94.5)	69 (42–103)	0.812
Serum ALT (UI/L)*	97 (56.5–170)	85 (55–127)	0.268
BMI *	22.3 (21.1–24.5)	23.1 (21.2–25.2)	0.495
Glycemia (mg/dL) *	89 (82–93)	89 (83–97.7)	0.740
Insulinemia (UI/L) *	12.4 (7.1–20.3)	13.4 (9.7–21.2)	0.713
Alcol (gr/die) *	0 (0–20)	0 (0–5)	0.611
HOMA index *	2.3 (1.5–3.8)	3.1 (1.8–4.6)	0.190

**LEGEND.** Data are presented as *median, (IQR) and °absolute number, (%). Differences between groups were compared by *Mann Whitney U test and °χ2 test. N-SVR, Non Sustained Virological Response: serum HCV-RNA (≥50 IU/mL) 24 weeks after the end of a full course of 48 or 72 weeks of anti-HCV treatment. SVR, Sustained Virological Response: undetectable serum HCV-RNA <50 UI/mL 24 weeks after the end of a full course of 48 or 72 weeks of anti-HCV treatment. NRTI, Nucleoside Reverse Transcriptase Inhibitors; NNRTI, Non Nucleoside Reverse Transcriptase Inhibitors; PI, Protease Inhibitors; MSM, men who have sex with men; WSM, women who have sex with men; IDUs, injection drug users; HCV, hepatitis C virus; HBV, hepatitis B virus; HBsAg, hepatitis B surface antigen; AST, Aspartate Aminotransferase; ALT, Alanine Aminotransferase; BMI, Body Mass Index. HOMA index, Homeostatic Model Assessment index.

Similarly to EVR patients, SVR subjects were characterized by a baseline milder liver disease: in fact, in comparison with N-SVR patients, they showed lower baseline median HCV viremia (HCV-RNA, 5.3 log_10_ cp/mL, IQR: 4.7–5.9 *versus* 5.68 log_10_ cp/mL, IQR: 5.3–6.2; p = 0.014) and they more rarely presented cirrhosis (9/41, 22% *vs* 19/44, 43%, p = 0.037). In the same way, HCV genotypes 1–4 were more represented in N-SVR patients (SVR 7, 17% - N-SVR 33, 70%, p = 0.0001) (Table 3).

We then investigated the predictive value of EVR on SVR in our patients: the positive predictive value of EVR for the achievement of SVR was 64.1% (41 EVR patients who reached SVR/64 EVR patients who reached the end of the study), on the contrary the negative predictive value was 100% (all 24 NR did not show sustained virological response).

### T cell activation, MT and host response to MT according to SVR to anti-HCV treatment

Peripheral T lymphocytes activation was similar in both groups (HLA-DR/CD38+/CD4+, SVR 30.8 (IQR 18.1–39.2) – N-SVR 25.3 (IQR 15.5–54), p = 0.933; HLA-DR/CD38+/CD8+, SVR 41 (IQR 29.3–56.6) – N-SVR 40 (IQR 26.5–62.5), p = 0.855) (Figure 3c, d).

On the contrary, SVR patients were characterized by lower sCD14 and LPS levels, reaching statistical significance for sCD14 (median, IQR: sCD14 SVR 2.61, 2.14–3.33 – N-SVR 3.11, 2.49–3.97, p = 0.026; LPS SVR 173.9, 151.6–243.9 – N-SVR 232.9, 148.7–330.3, p = 0.164) (figure 4c, d).

### Identification of predictors of SVR to anti-HCV treatment

To investigate the possible predictive value of MT on SVR, we conducted a logistic regression analysis including HIV and HCV related variables and both LPS and sCD14 (Table 4).

**Table 4:** Association between markers of microbial translocation and Sustained Virological Response to anti-HCV treatment.

	Univariate			Multivariate		
	OR	95%CI	p	AOR	95%CI	p
LPS (pg/mL)	0.996	0.990–1.001	0.106	1.000	0.980–1.003	0.129
sCD14 (µg/mL)	0.668	0.428–1.041	**0.046**	0.584	0.214–1.589	0.292
HCV genotypes(1–4 vs 2–3)	0.087	0.031–0.244	**0.0001**	0.022	0.001–0.469	**0.014**
HCV-RNA(log_10_ IU/mL)	0.423	0.224–0.798	**0.008**	0.778	0.309–10.231	0.519
Fibrosis(advanced vs non advanced)	0.498	0.200–1.194	0.116	0.553	0.026–11.663	0.703
Cirrhosis (yes vs no)	0.370	0.143–0.957	**0.040**	0.161	0.007–4.472	0.289
Nadir CD4+ T cells/µL	1.003	1.000–1.006	0.071	1.005	0.997–1.014	0.835
CD4+ T cells/µL	1.001	0.999–1.003	0.287	1.000	0.995–1.009	0.669
Age, years	0.996	0.908–1.093	0.996	0.942	0.719–1.236	0.870
Sex, male vs female	0.722	0.237–2.200	0.566	0.812	0.067–9.871	0.216

**LEGEND.** LPS, soluble CD14, CD4+ T cells/µL, age, HCV-RNA log_10_ cp/mL for each unit more. sCD14 and LPS were measured in plasma samples; sCD14 µg/mL, LPS pg/mL. Multivariate analysis was performed in 65/98 patients for whom all the clinical, epidemiological and biological parameters under study were available.

OR, odds ratio; AOR, adjusted odds ratio; CI, confidence interval. p>0.05 was considered non significant.

Interestingly, in the univariate model, higher circulating sCD14 and LPS are associated with a reduced chance of SVR, reaching significance for sCD14 (OR 0.668 for each sCD14 unit more, 95%CI 0.428–1.041, p = 0.046; OR 0.996 for each LPS unit more, 95%CI 0.990–1.001, p = .106). In addition, several HCV parameters were related to SVR: HCV genotypes 1–4 (OR 0.087 *versus* HCV genotypes 2–3, 95%CI 0.031–0.244, p = 0.0001), HCV viral load (OR 0.423 for each log_10_ IU/mL more, 95%CI 0.224–0.798, p = 0.008), and cirrhosis (OR 0.37 *versus* absence of cirrhosis, 95%CI 0.143–0.957, p = 0.04) were predictive of a reduced probability of SVR in the univariate model.

In line with univariate data, the proportion of patients with cirrhosis were more likely to fail SVR (cirrhosis/N-SVR = 43%, cirrhosis/SVR = 22%, p = 0.037); similarly a higher proportion of genotype 1–4 patients did not reach SVR (genotype 1–4/N-SVR = 70%, genotype 1–4/SVR = 17%, p = 0.0001). No differences were shown in SVR/N-SVR according to the presence fibrosis (p = 0.114).

We next performed multivariate analysis in 65/98 patients for whom both LPS and sCD14 data were available (Table 4). Baseline characteristics of these 65 patients were comparable to the entire cohort. In multivariate logistic regression, the most important predictive parameter of SVR was HCV genotype (AOR 0.022 HCV genotypes 1–4 *versus* 2–3, 95%CI 0.001–0.469, p = 0.014). No further measures of MT and immune response to MT that we measured were independently associated with SVR (Table 4).

Similar results were obtained also excluding from the analyses the 8/98 patients who were lost at follow up before 12 weeks (data not shown).

Results were similar in a sensitivity analysis including only HIV/HCV co-infected patients on HAART (n = 96): despite comparable LPS, EVR patients presented lower sCD14 than NR (median, IQR: sCD14 EVR 2.74, 2.18–3.26 – NR 3.75, 2.89–4.15, p = 0.0001; LPS EVR 178.7, 150.7–298.3 – NR 195.8, 75–321.9, p = 0.649 (Figure S1a, b).

Accordingly, lower sCD14 was displayed by SVR as compared to N-SVR (median, IQR: sCD14 SVR 2.63, 2.15–3.37 – N-SVR 3.06, 2.48–3.87, p = 0.041; LPS SVR 173.9, 151.6–243.9 – N-SVR 214.1, 145.3–329.5, p = 0.213) (Figure S1c, d).

Furthermore, sCD14 confirmed independently associated with EVR (Tables S1 and S2).

## Discussion

In cART-treated HIV/HCV co-infected patients we show: (i) greater host responsiveness to LPS in patients with most aggressive HCV-related liver disease and harboring HCV genotypes 1–4; (ii) low levels of LPS/sCD14 as predictors of virological response to 3 months of anti-HCV treatment (i.e. EVR).

Response to anti-HCV therapy is less satisfactory in HIV/HCV-co-infected individuals, urging the identification of predictive outcome markers.

There is increasing evidence that MT and MT-driven immune activation are pathogenetic mechanisms of alcohol-driven liver disease [35], [36], and of hepatic disease progression in HBV and HCV mono-infection as well as in HIV/HCV co-infection [27], [33].

In line with this hypothesis, our data show that HIV/HCV co-infected patients with cirrhosis display higher circulating sCD14 that is a marker of MT-driven immune activation [24], [27].

Having shown the association between heightened circulating sCD14 and most advanced liver disease, we next aimed to investigate whether MT and the host response that it elicits could condition short- and long- term virological outcome to anti-HCV treatment.

Our major finding is that, despite equal MT in HIV/HCV co-infected patients with and without EVR, a contained host response to translocated microbial by-products as measured by low levels of circulating sCD14, independently predicts EVR.

As for HCV-related factors, only HCV genotype was confirmed to independently affect response to antiviral treatment. Although the effect of HCV genotypes on HCV/HIV pathogenesis, disease progression and response to therapy are largely unknown, genotypes 1–4 are commonly defined as difficult-to-treat given the much lower probability of response to conventional HCV therapy [37], [38]. Interestingly, patients displaying HCV genotypes 1–4 also presented elevated sCD14 levels. This association remained significant also excluding from the analysis patients characterized by severe forms of liver disease, suggesting that difficult-to-treat HCV genotypes might *per se* associate to heightened immune activation, possibly due to MT.

Immune activation has been suggested to accelerate liver disease in HIV/HCV co-infection, in turn limiting response to therapy [13], [39], [40]. In our study, activated HLA-DR+CD38+ T cells were increased in comparison with levels of HIV mono-infected patients and healthy subjects reported in literature [41], and yet they failed significant association with HCV genotypes, liver disease and virological response to anti-HCV treatment.

On the contrary, by showing higher sCD14 levels in NR patients, our data indicate enhanced activation of innate immunity as possible correlate of failing response to therapy, that might be secondary to increased MT.

However, should MT be indeed a relevant pathway in conditioning anti-HCV response through its effect in triggering excessive immune activation, one would expect a parallel expansion in markers of cellular and innate immune activation [24], [42]. At least two non-mutually exclusive reasons might explain the seemingly discrepant trend in activated CD8+ and sCD14 levels. A first consideration is that the large majority of our patients were on long-term HAART with undetectable HIV-RNA, that is a known major determinant of T cell activation [43]. Secondly, HCV infection triggers immune activation mainly in monocytes and liver-residing macrophages, the activation of which sustains liver inflammation [40]. Given the role of hepatic Kupffer cells in detoxification of bloodstream bacterial components, high levels of microbial products in the bloodstream and in the liver with its consequent down-stream effects can be one of the mechanisms involved in reduced control of virus replication after anti-HCV treatment. Therefore, a unique pro-inflammatory/activated *milieu* might be speculated within the liver that fails to be detected by means of peripheral blood investigation. According to this model, Sandler *et al*. recently described high density of CD14+CD68+ in the liver correlating with hepatic disease progression, strongly suggestive for LPS-driven activation of hepatic Kupffer cells [27].

Our findings suggest that the host response to increased MT might play a central role in the pathogenesis of HIV/HCV-related liver injury, and in the inappropriate response to therapy, allowing to speculate a vicious cycle, whereby HIV effects on the gut mucosa might enhance MT which stimulate hepatic fibrosis/cirrhosis with consequent portal systemic shunt, to further fuel MT.

However, when we investigated the independent effect of MT on long-term response to therapy, sCD14 was still associated to SVR in the univariate analysis, but lost independent predictive value in the multivariable model, suggesting that immune response to MT might play a role in the earlier phases of treatment. Because EVR conditions SVR, this could underline the importance of assessing (and controlling) MT before starting anti-HCV therapy.

In the present study, we analysed only circulating sCD14 as marker of monocyte activation, and as possible reflection of MT. The investigation of additional markers of monocyte activation (e.g. soluble CD163) and systemic inflammation (e.g. IL-6) will help gain a broader definition of the effect of systemic immune activation/inflammation on HCV disease progression and response to therapy.

As alternative hypothesis, increased sCD14 in patients with worst liver disease and HCV genotype 1–4 might reflect heightened endogenous interferon (IFN) rather than increased MT. Indeed, HCV infection activates the endogenous IFN system in the liver [44]; yet such gene pre-activation is associated to the lack of response to IFN-alpha/ribavirin, possibly through a refractory state to IFN-mediated signalling [45]–[48]. Patients harbouring HCV genotypes 1–4 have a higher likelihood of failing EVR/SVR and have higher levels of endogenous IFN and IFN-stimulated gene (ISG) expression [18], [49]. Given that the exposure to IFN has been shown to transiently activate macrophages with sCD14 release [50], and that CD14+ monocytes of HIV-infected patients display higher ISG expression [51], our data that patients with cirrhosis, 1–4 genotypes and non-responders show increased sCD14 despite equal plasma LPS, allow to hypothesise in these individuals an overall higher innate immune activation with higher endogenous IFN, that results in sCD14 release, while rendering the cells less sensitive to exogenous IFN.

Some limitations of our study need to be discussed. Our research focused on HIV/HCV co-infected patients. However, given that hepatocytes are a major source of sCD14, a control group of HCV mono-infected individuals would help establish cause-effect nexus between the host response to MT and severity of liver disease and response to therapy in the setting of HCV/HIV co-infection.

Nevertheless, despite our study was not specifically designed to establish causality, it well adds to the debate on the role of microbial translocation and its downstream effects of immune activation in the setting of HCV infection and disease. In particular, our findings expands on data by both by Sandler *et al*. in HCV mono-infected patients showing elevated circulating sCD14 as independent predictor of end-stage liver disease [27], and Balagopal *et al.* in HIV/HCV co-infected patients correlating elevated sCD14/LPS with cirrhosis [33], as they strongly suggest an independent influence of immune activation due to MT in the outcome of anti-HCV treatment.

A further limitation of our research is that, despite associating with EVR, augmented sCD14 seemingly fail to influence sustained response to therapy, in turn limiting its exploitation in the clinical practice as measure to *a priori* include/exclude patients from treatment.

Knowing the prognostic significance of EVR for SVR, larger studies are needed to shed light on MT in HIV/HCV co-infection pathogenesis and response to therapy, essential premise to evaluate the possibility of novel therapeutic adjuvant interventions targeting translocation of bacterial products in peripheral blood ant its downstream effects.

## Supporting Information

Figure S1 **Circulating sCD14 and LPS levels are higher in NR and in N-SVR patients on HAART.** Results from a sensitivity analysis including only HIV/HCV co-infected patients on HAART (n = 96) are shown. **a**)**-b**) sCD14 and LPS were compared between patients with early virological response [EVR, i.e. undetectable serum HCV-RNA (<50 IU/mL) or ≥2 log_10_ reduction from baseline after 12 weeks of therapy], and Null Responders (NR) (i.e. serum HCV-RNA ≥50 IU/mL and <2 log_10_ reduction from baseline). **c**)**-d**) sCD14 and LPS were compared between patients with sustained virological response [SVR, i.e. undetectable serum HCV-RNA (<50 IU/mL) 24 weeks after the end of a full course of 48 or 72 weeks of anti-HCV treatment, according to genotype], and N-SVR subjects.
(TIF)

Table S1 **Association between markers of microbial translocation and Early Virological Response to anti-HCV treatment on patients on HAART.** Univariate and multivariate logistic regression conducted including only HIV/HCV patients on HAART (n 96) to explore association between markers of microbial translocation (sCD14 and LPS) and EVR. The multivariate analysis is adjusted for demographic, HCV- and HIV-related variables. LPS, soluble CD14, CD4+ T cells/µL, age, HCV-RNA log_10_ cp/mL for each unit more. sCD14 and LPS were measured in plasma samples; sCD14 µg/mL, LPS pg/mL. OR, odds ratio; AOR, adjusted odds ratio; CI, confidence interval. p>0.05 was considered non significant.
(DOC)

Table S2 **Association between markers of microbial translocation and Sustained Virological Response to anti-HCV treatment on patients on HAART.** Univariate and multivariate logistic regression conducted including only HIV/HCV patients on HAART (n 96) to explore association between markers of microbial translocation (sCD14 and LPS) and SVR. The multivariate analysis is adjusted for demographic, HCV- and HIV-related variables. LPS, soluble CD14, CD4+ T cells/µL, age, HCV-RNA log_10_ cp/mL for each unit more. sCD14 and LPS were measured in plasma samples; sCD14 µg/mL, LPS pg/mL. OR, odds ratio; AOR, adjusted odds ratio; CI, confidence interval. p>0.05 was considered non significant.
(DOC)

## References

[1] Rockstroh JK, Mocroft A, Soriano V, Tural C, Losso MH (2005). Influence of hepatitis C virus infection on HIV-1 disease progression and response to highly active antiretroviral therapy.. J Infect Dis.

[2] Soriano V, Mocroft A, Rockstroh J, Ledergerber B, Knysz B (2008). Spontaneous viral clearance, viral load, and genotype distribution of hepatitis C virus (HCV) in HIV-infected patients with anti-HCV antibodies in Europe.. J Infect Dis.

[3] Bica I, McGovern B, Dhar R, Stone D, McGowan K (2001). Increasing mortality due to end-stage liver disease in patients with human immunodeficiency virus infection.. Clin Infect Dis.

[4] Graham CS, Baden LR, Yu E, Mrus JM, Carnie J (2001). Influence of human immunodeficiency virus infection on the course of hepatitis C virus infection: a meta-analysis.. Clin Infect Dis.

[5] Rosenthal E, Salmon-Céron D, Lewden C, Bouteloup V, Pialoux G (2009). Liver-related deaths in HIV-infected patients between 1995 and 2005 in the French GERMIVIC Joint Study Group Network (Mortavic 2005 study in collaboration with the Mortalité 2005 survey, ANRS EN19).. HIV Med.

[6] Puoti M, Prestini K, Putzolu V, Zanini B, Baiguera C (2003). HIV/HCV co-infection: natural history.. J Biol Regul Homeost Agents.

[7] Bruno R, Sacchi P, Puoti M, Soriano V, Filice G (2002). HCV chronic hepatitis in patients with HIV: clinical management issues.. Am J Gastroenterol.

[8] Bruno R, Sacchi P, Puoti M, Maiocchi L, Patruno S (2007). Natural history of compensated viral cirrhosis in a cohort of patients with HIV infection.. J Acquir Immune Defic Syndr.

[9] Blackard JT, Sherman KE (2008). HCV/HIV co-infection: time to re-evaluate the role of HIV in the liver?. J Viral Hepat.

[10] Rockstroh JK (2006). Influence of viral hepatitis on HIV infection.. J Hepatol.

[11] Berzsenyi MD, Bowden DS, Kelly HA, Watson KM, Mijch AM (2007). Reduction in hepatitis C-related liver disease associated with GB virus C in human immunodeficiency virus coinfection.. Gastroenterology.

[12] McGovern BH (2007). Hepatitis C in the HIV-infected patient.. J Acquir Immune Defic Syndr.

[13] Gonzalez VD, Falconer K, Blom KG, Reichard O, Mørn B (2009). High levels of chronic immune activation in the T-cell compartments of patients coinfected with hepatitis C virus and human immunodeficiency virus type 1 and on highly active antiretroviral therapy are reverted by alpha interferon and ribavirin treatment.. J Virol.

[14] Ge D, Fellay J, Thompson AJ, Simon JS, Shianna KV (2009). Genetic variation in IL28B predicts hepatitis C treatment-induced viral clearance.. Nature.

[15] Rauch A, Kutalik Z, Descombes P, Cai T, Di Iulio J (2010). Genetic variation in IL28B is associated with chronic hepatitis C and treatment failure: a genome-wide association study.. Gastroenterology.

[16] Suppiah V, Moldovan M, Ahlenstiel G, Berg T, Weltman M (2009). IL28B is associated with response to chronic hepatitis C interferon-alpha and ribavirin therapy.. Nat Genet.

[17] Tanaka Y, Nishida N, Sugiyama M, Kurosaki M, Matsuura K (2009). Genome-wide association of IL28B with response to pegylated interferon-alpha and ribavirin therapy for chronic hepatitis C.. Nat Genet.

[18] Dill MT, Duong FH, Vogt JE, Bibert S, Bochud PY (2011). Interferon-induced gene expression is a stronger predictor of treatment response than IL28B genotype in patients with hepatitis C.. Gastroenterology.

[19] Yu JW, Wang GQ, Sun LJ, Li XG, Li SC (2007). Predictive value of rapid virological response and early virological response on sustained virological response in HCV patients treated with pegylated interferon alpha-2a and ribavirin.. J Gastroenterol Hepatol.

[20] Elefsiniotis IS, Vezali E, Mihas C, Saroglou G (2009). Predictive value of complete and partial early virological response on sustained virological response rates of genotype-4 chronic hepatitis C patients treated with PEG-interferon plus ribavirin.. Intervirology.

[21] Wyles DL (2010). Moving beyond interferon alfa: investigational drugs for hepatitis C virus infection.. Top HIV Med.

[22] Vermehren J, Sarrazin C (2011). New HCV therapies on the horizon.. Clin Microbiol Infect.

[23] Mattapallil JJ, Douek DC, Hill B, Nishimura Y, Martin M (2005). Massive infection and loss of memory CD4+ T cells in multiple tissues during acute SIV infection.. Nature.

[24] Brenchley JM, Price DA, Schacker TW, Asher TE, Silvestri G (2006). Microbial translocation is a cause of systemic immune activation in chronic HIV infection.. Nat Med.

[25] Marchetti G, Cozzi-Lepri A, Merlini E, Bellistrì GM, Castagna A (2011). Microbial translocation predicts disease progression of HIV-infected antiretroviral-naive patients with high CD4+ cell count.. AIDS.

[26] Jiang W, Lederman MM, Hunt P, Sieg SF, Haley K (2009). Plasma levels of bacterial DNA correlate with immune activation and the magnitude of immune restoration in persons with antiretroviral-treated HIV infection.. J Infect Dis.

[27] Sandler NG, Koh C, Roque A, Eccleston JL, Siegel RB (2011). Host response to translocated microbial products predicts outcomes of patients with HBV or HCV infection.. Gastroenterology.

[28] Dolganiuc A, Norkina O, Kodys K, Catalano D, Bakis G (2007). Viral and host factors induce macrophage activation and loss of toll-like receptor tolerance in chronic HCV infection.. Gastroenterology.

[29] Gaeta GB, Perna P, Adinolfi LE, Utili R, Ruggiero G (1982). Endotoxemia in a series of 104 patients with chronic liver diseases: prevalence and significance.. Digestion.

[30] Bode C, Kugler V, Bode JC (1987). Endotoxemia in patients with alcoholic and non-alcoholic cirrhosis and in subjects with no evidence of chronic liver disease following acute alcohol excess.. J Hepatol.

[31] Harte AL, da Silva NF, Creely SJ, McGee KC, Billyard T (2010). Elevated endotoxin levels in non-alcoholic fatty liver disease.. J Inflamm (Lond).

[32] Caradonna L, Mastronardi ML, Magrone T, Cozzolongo R, Cuppone R (2002). Biological and clinical significance of endotoxemia in the course of hepatitis C virus infection.. Curr Pharm Des.

[33] Balagopal A, Philp FH, Astemborski J, Block TM, Mehta A (2008). Human immunodeficiency virus-related microbial translocation and progression of hepatitis C.. Gastroenterology.

[34] Castera L, Forns X, Alberti A (2008). Non-invasive evaluation of liver fibrosis using transient elastography.. J Hepatol.

[35] Thurman RG (1998). II. Alcoholic liver injury involves activation of Kupffer cells by endotoxin.. Am J Physiol.

[36] Enomoto N, Yamashina S, Kono H, Schemmer P, Rivera CA (1999). Development of a new, simple rat model of early alcohol-induced liver injury based on sensitization of Kupffer cells.. Hepatology.

[37] Farnik H, Mihm U, Zeuzem S (2009). Optimal therapy in genotype 1 patients.. Liver Int.

[38] Kamal SM (2009). Hepatitis C genotype 4 therapy: increasing options and improving outcomes.. Liver Int.

[39] Kovacs A, Karim R, Mack WJ, Xu J, Chen Z (2010). Activation of CD8 T cells predicts progression of HIV infection in women coinfected with hepatitis C virus.. J Infect Dis.

[40] Gonzalez VD, Landay AL, Sandberg JK (2010). Innate immunity and chronic immune activation in HCV/HIV-1 co-infection.. Clin Immunol.

[41] Hunt PW, Martin JN, Sinclair E, Bredt B, Hagos E (2003). T cell activation is associated with lower CD4+ T cell gains in human immunodeficiency virus-infected patients with sustained viral suppression during antiretroviral therapy.. J Infect Dis.

[42] Funderburg N, Luciano AA, Jiang W, Rodriguez B, Sieg SF (2008). Toll-like receptor ligands induce human T cell activation and death, a model for HIV pathogenesis.. PLoS One.

[43] Deeks SG, Kitchen CM, Liu L, Guo H, Gascon R (2004). Immune activation set point during early HIV infection predicts subsequent CD4+ T-cell changes independent of viral load.. Blood.

[44] Thomas DL, Thio CL, Martin MP, Qi Y, Ge D (2009). Genetic variation in IL28B and spontaneous clearance of hepatitis C virus.. Nature.

[45] Sarasin-Filipowicz M, Oakeley EJ, Duong FH, Christen V, Terracciano L (2008). Interferon signaling and treatment outcome in chronic hepatitis C.. Proc Natl Acad Sci U S A.

[46] Chen L, Borozan I, Feld J, Sun J, Tannis LL (2005). Hepatic gene expression discriminates responders and nonresponders in treatment of chronic hepatitis C viral infection.. Gastroenterology.

[47] Asselah T, Bieche I, Narguet S, Sabbagh A, Laurendeau I (2008). Liver gene expression signature to predict response to pegylated interferon plus ribavirin combination therapy in patients with chronic hepatitis C.. Gut.

[48] Sarasin-Filipowicz M, Wang X, Yan M, Duong FH, Poli V (2009). Alpha interferon induces long-lasting refractoriness of JAK-STAT signaling in the mouse liver through induction of USP18/UBP43.. Mol Cell Biol.

[49] Urban TJ, Thompson AJ, Bradrick SS, Fellay J, Schuppan D (2010). IL28B genotype is associated with differential expression of intrahepatic interferon-stimulated genes in patients with chronic hepatitis C.. Hepatology.

[50] Brettschneider J, Ecker D, Bitsch A, Bahner D, Bogumil T (2002). The macrophage activity marker sCD14 is increased in patients with multiple sclerosis and upregulated by interferon beta-1b.. J Neuroimmunol.

[51] Rempel H, Sun B, Calosing C, Pillai SK, Pulliam L (2010). Interferon-alpha drives monocyte gene expression in chronic unsuppressed HIV-1 infection.. AIDS.

