# Supplementary figures and images for: Circulating sCD14 Is Associated with Virological Response to Pegylated-Interferon-Alpha/Ribavirin Treatment in HIV/HCV Co-Infected Patients
Source: PLoS One. 2012 Feb 21;7(2):e32028. doi: 10.1371/journal.pone.0032028 (PMC3283684; doi:10.1371/journal.pone.0032028)

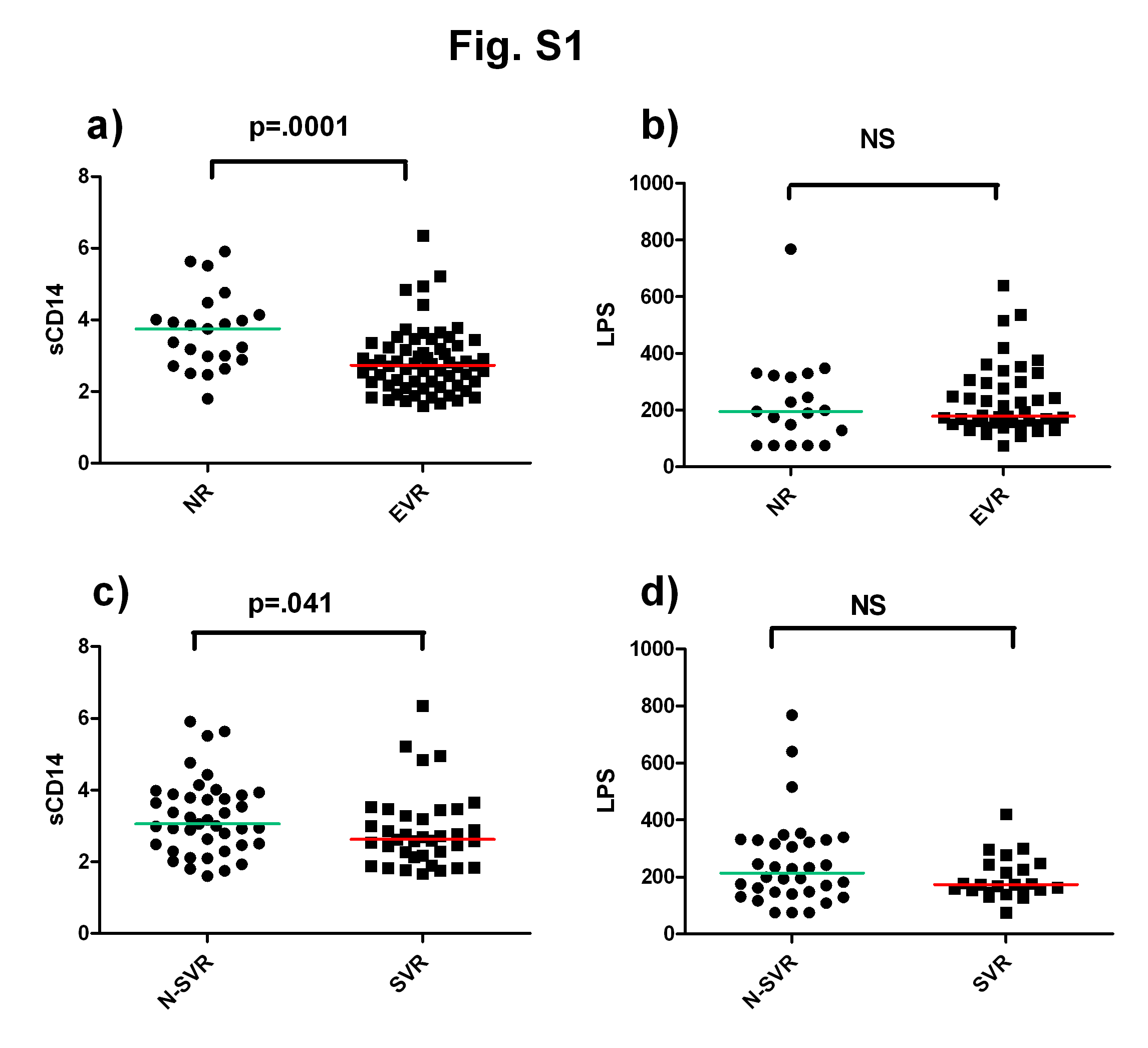

Supplement: Figure S1 — Circulating sCD14 and LPS levels are higher in NR and in N-SVR patients on HAART. Results from a sensitivity analysis including only HIV/HCV co-infected patients on HAART (n = 96) are shown. a)-b) sCD14 and LPS were compared between patients with early virological response [EVR, i.e. undetectable serum HCV-RNA (<50 IU/mL) or ≥2 log10 reduction from baseline after 12 weeks of therapy], and Null Responders (NR) (i.e. serum HCV-RNA ≥50 IU/mL and <2 log10 reduction from baseline). c)-d) sCD14 and LPS were compared between patients with sustained virological response [SVR, i.e. undetectable serum HCV-RNA (<50 IU/mL) 24 weeks after the end of a full course of 48 or 72 weeks of anti-HCV treatment, according to genotype], and N-SVR subjects. (TIF) [file pone.0032028.s001.tif]
