# Supplementary material for: Circulating sCD14 Is Associated with Virological Response to Pegylated-Interferon-Alpha/Ribavirin Treatment in HIV/HCV Co-Infected Patients
Source: PLoS One. 2012 Feb 21;7(2):e32028. doi: 10.1371/journal.pone.0032028 (PMC3283684; doi:10.1371/journal.pone.0032028)
Supplement: Table S1 — Association between markers of microbial translocation and Early Virological Response to anti-HCV treatment on patients on HAART. Univariate and multivariate logistic regression conducted including only HIV/HCV patients on HAART (n 96) to explore association between markers of microbial translocation (sCD14 and LPS) and EVR. The multivariate analysis is adjusted for demographic, HCV- and HIV-related variables. LPS, soluble CD14, CD4+ T cells/µL, age, HCV-RNA log10 cp/mL for each unit more. sCD14 and LPS were measured in plasma samples; sCD14 µg/mL, LPS pg/mL. OR, odds ratio; AOR, adjusted odds ratio; CI, confidence interval. p>0.05 was considered non significant. (DOC) [file pone.0032028.s002.doc]

**Table S1**

Association between markers of microbial translocation and Early Virological Response to anti-HCV treatment on patients on HAART.

|  | **Univariate** | | | **Multivariate** | | |
| --- | --- | --- | --- | --- | --- | --- |
|  | **OR** | **95%CI** | **p** | **AOR** | **95%CI** | **p** |
| LPS (pg/mL) | 1.000 | 0.996-1.005 | 0.854 | 0.997 | 0.99-1.004 | 0.345 |
| sCD14 (μg/mL) | 0.440 | 0.263-0.738 | **0.002** | 0.145 | 0.031-0.688 | **0.015** |
| HCV genotypes  (1-4 vs 2-3) | 0.117 | 0.039-0.348 | **0.0001** | 0.233 | 0.021-2.618 | 0.238 |
| HCV-RNA  (log_10_ IU/mL) | 0.394 | 0.198-0.787 | **0.008** | 0.789 | 0.134-4.628 | 0.793 |
| Fibrosis  (advanced vs non advanced) | 0.486 | 0.184-1.284 | 0.146 | 0.134 | 0.005-3.879 | 0.616 |
| Cirrhosis (yes vs no) | 0.394 | 0.149-1.042 | 0.06 | 0.185 | 0.007-4.623 | 0.304 |
| Nadir CD4+ T cells/μL | 1.003 | 0.999-1.006 | 0.195 | 1.007 | 0.998-1.016 | 0.134 |
| CD4+ T cells/μL | 0.999 | 0.997-1.001 | 0.439 | 0.996 | 0.990-1.001 | 0.112 |
| Age, years | 1.035 | 0.938-1.142 | 0.489 | 1.134 | 0.879-1.463 | 0.333 |
| Sex, male vs female | 0.572 | 0.149-2.196 | 0.416 | 0.215 | 0.007-6.926 | 0.385 |

LPS, soluble CD14, CD4+ T cells/μL, age, HCV-RNA log_10_ cp/mL for each unit more.

sCD14 and LPS were measured in plasma samples; sCD14 μg/mL, LPS pg/mL.

OR, odds ratio; AOR, adjusted odds ratio; CI, confidence interval. p>0.05 was considered non significant.
