# Supplementary material for: Circulating sCD14 Is Associated with Virological Response to Pegylated-Interferon-Alpha/Ribavirin Treatment in HIV/HCV Co-Infected Patients
Source: PLoS One. 2012 Feb 21;7(2):e32028. doi: 10.1371/journal.pone.0032028 (PMC3283684; doi:10.1371/journal.pone.0032028)
Supplement: Table S2 — Association between markers of microbial translocation and Sustained Virological Response to anti-HCV treatment on patients on HAART. Univariate and multivariate logistic regression conducted including only HIV/HCV patients on HAART (n 96) to explore association between markers of microbial translocation (sCD14 and LPS) and SVR. The multivariate analysis is adjusted for demographic, HCV- and HIV-related variables. LPS, soluble CD14, CD4+ T cells/µL, age, HCV-RNA log10 cp/mL for each unit more. sCD14 and LPS were measured in plasma samples; sCD14 µg/mL, LPS pg/mL. OR, odds ratio; AOR, adjusted odds ratio; CI, confidence interval. p>0.05 was considered non significant. (DOC) [file pone.0032028.s003.doc]

**Table S2**

Association between markers of microbial translocation and Sustained Virological Response to anti-HCV treatment on patients on HAART.

|  | **Univariate** | | | **Multivariate** | | |
| --- | --- | --- | --- | --- | --- | --- |
|  | **OR** | **95%CI** | **p** | **AOR** | **95%CI** | **p** |
| LPS (pg/mL) | 0.996 | 0.991-1.001 | 0.136 | 0.991 | 0.980-1.003 | 0.129 |
| sCD14 (μg/mL) | 0.700 | 0.445-1.103 | 0.124 | 0.584 | 0.214-1.589 | 0.292 |
| HCV genotypes  (1-4 vs 2-3) | 0.093 | 0.033-0.260 | **0.0001** | 0.022 | 0.001-0.464 | **0.014** |
| HCV-RNA  (log_10_ IU/mL) | 0.394 | 0.205-0.760 | **0.005** | 0.778 | 0.309-10.231 | 0.519 |
| Fibrosis  (advanced vs non advanced) | 0.457 | 0.185-1.126 | 0.089 | 0.553 | 0.026-11.663 | 0.703 |
| Cirrhosis (yes vs no) | 0.347 | 0.130-0.928 | **0.035** | 0.171 | 0.007-4.472 | 0.289 |
| Nadir CD4+ T cells/μL | 1.003 | 0.999-1.006 | 0.120 | 1.005 | 0.997-1.014 | 0.835 |
| CD4+ T cells/μL | 1.001 | 0.999-1.003 | 0.364 | 1.000 | 0.995-1.009 | 0.669 |
| Age, years | 0.995 | 0.904-1.094 | 0.911 | 0.942 | 0.719-1.236 | 0.870 |
| Sex, male vs female | 0.846 | 0.269-2.661 | 0.775 | 0.812 | 0.067-9.871 | 0.216 |

LPS, soluble CD14, CD4+ T cells/μL, age, HCV-RNA log_10_ cp/mL for each unit more.

sCD14 and LPS were measured in plasma samples; sCD14 μg/mL, LPS pg/mL.

OR, odds ratio; AOR, adjusted odds ratio; CI, confidence interval. p>0.05 was considered non significant
